# A Mango Leaf Extract (Zynamite^®^) Combined with Quercetin Has Exercise-Mimetic Properties in Human Skeletal Muscle

**DOI:** 10.3390/nu15132848

**Published:** 2023-06-23

**Authors:** Miriam Martinez-Canton, Victor Galvan-Alvarez, Eduardo Garcia-Gonzalez, Angel Gallego-Selles, Miriam Gelabert-Rebato, Giovanni Garcia-Perez, Alfredo Santana, Laura Lopez-Rios, Tanausu Vega-Morales, Marcos Martin-Rincon, Jose A. L. Calbet

**Affiliations:** 1 Department of Physical Education and Research Institute of Biomedical and Health Sciences (IUIBS), University of Las Palmas de Gran Canaria, Campus Universitario de Tafira s/n, 35017 Las Palmas de Gran Canaria, Spain; miriammartinezcanton@gmail.com (M.M.-C.); victor_galvan@hotmail.es (V.G.-A.); eduardo.garcia124@alu.ulpgc.es (E.G.-G.); angelgallegoselles@hotmail.com (A.G.-S.); miriamgela@hotmail.com (M.G.-R.); giovannigarcia94perez@gmail.com (G.G.-P.); consultageneticasantana@gmail.com (A.S.); 2 Clinical Genetics Unit, Complejo Hospitalario Universitario Insular-Materno Infantil de Las Palmas de Gran Canaria, 35016 Las Palmas de Gran Canaria, Spain; 3 Nektium Pharma, Las Mimosas 8, Agüimes, 35118 Las Palmas de Gran Canaria, Spain; llopez@nektium.com (L.L.-R.); tvega@nektium.com (T.V.-M.); 4 Department of Physical Performance, Norwegian School of Sport Sciences, 0806 Oslo, Norway

**Keywords:** ergogenic aids, signaling, antioxidant supplementation, high-intensity exercise, ischaemia-reperfusion, sports nutrition, polyphenols, muscle function, human

## Abstract

Zynamite PX^®^, a mango leaf extract combined with quercetin, enhances exercise performance by unknown molecular mechanisms. Twenty-five volunteers were assigned to a control (17 males) or supplementation group (8 males, receiving 140 mg of Zynamite^®^ + 140 mg quercetin/8 h for 2 days). Then, they performed incremental exercise to exhaustion (IE) followed by occlusion of the circulation in one leg for 60 s. Afterwards, the cuff was released, and a 30 s sprint was performed, followed by 90 s circulatory occlusion (same leg). *Vastus lateralis* muscle biopsies were obtained at baseline, 20 s after IE (occluded leg) and 10 s after Wingate (occluded leg), and bilaterally at 90 s and 30 min post exercise. Compared to the controls, the Zynamite PX^®^ group showed increased basal protein expression of Thr^287^-CaMKIIδ_D_ (2-fold, *p* = 0.007) and Ser^9^-GSK3β (1.3-fold, *p* = 0.005) and a non-significant increase of total NRF2 (1.7-fold, *p* = 0.099) and Ser^40^-NRF2 (1.2-fold, *p* = 0.061). In the controls, there was upregulation with exercise and recovery of total NRF2, catalase, glutathione reductase, and Thr^287^-CaMKIIδ_D_ (1.2–2.9-fold, all *p* < 0.05), which was not observed in the Zynamite PX^®^ group. In conclusion, Zynamite PX^®^ elicits muscle signaling changes in resting skeletal muscle resembling those described for exercise training and partly abrogates the stress kinases responses to exercise as observed in trained muscles.

## 1. Introduction

Natural polyphenols combine aromatic rings with a variety of functional groups, which entail a great diversity of physiological effects conferring antioxidant, cardioprotective, neuroprotective, anticancer, immunomodulatory, prebiotic, ergogenic (enhancement of exercise performance), and antimicrobial properties [1,2,3]. Zynamite^®^, an extract from mango leaves abundant in mangiferin, has been shown to enhance power output when combined with quercetin or luteolin [4,5]. Moreover, Zynamite^®^ accelerates recovery after exhausting exercise [3] and attenuates the negative effects of ischemia-reperfusion on muscle function [4,5,6]. Zynamite PX^®^, a polyphenolic extract combining the mango leaf extract with quercetin, has been shown to enhance exercise performance after a single dose [6], as well as after 48 h [4,5] and 15 days of supplementation [4,5]. Zynamite^®^ has also been shown to improve reaction time in humans and long-term potentiation in the hippocampus in rodents [7]. However, no previous study has determined whether Zynamite PX^®^ may trigger molecular changes in skeletal muscle analogous to the ones elicited by physical exercise, thus functioning as an exercise mimetic.

Mangiferin has iron-chelating capacity and remarkable antioxidant power due to its free-radical scavenging properties and its ability to inhibit nicotinamide adenine dinucleotide phosphate-oxidase (NADPH oxidase or NOX) and xanthine oxidase (XO), which are prominent producers of reactive oxygen species (ROS) in exercise [8] and inflammation [9,10,11,12,13,14]. Quercetin, which also has potent antioxidant and anti-inflammatory actions [15], may enhance aerobic exercise performance [16] and power output during sprint exercise when administered together with Zynamite^®^ [4,5]. Animal and cell culture experiments have shown that both mangiferin [13,17,18,19] and quercetin exert protection against damage caused by ischemia-reperfusion [20,21,22,23], which could, in part, be explained by their inhibitory effects on XO and NOX [24,25].

ROS production during exercise is more prominent during high-intensity and prolonged exercise, mainly if performed until exhaustion [8,26,27,28] or in severe hypoxia [29]. Although uncontrolled ROS production could cause oxidative damage and fatigue [30], exercise training increases skeletal muscle antioxidant capacity [31,32,33] and reduces ROS-induced signaling [8,27] and damage [34]. Part of the signaling responses needed for the adaptive response to exercise is mediated by ROS [26,35]. Although several enzymes and transcription factors are ROS-sensitive, the nuclear factor erythroid-derived 2-like 2 (NRF2) is the primary ROS sensor in most cells, including skeletal muscle [36,37,38,39]. Upon stimulation, NRF2 translocates to the nucleus, where it can interact with more than 250 genes possessing specific deoxyribonucleic acid (DNA) sequences called antioxidant response elements (AREs) involved in inflammation, autophagy, metabolism, mitochondrial biogenesis, detoxification, cytoprotection, cell differentiation, and the xenobiotic and antioxidant response [2]. Free NRF2 levels are regulated by Kelch-like ECH-associated protein 1 (Keap1), which under normal unstressed conditions, binds to NRF2, prevents its translocation to the nucleus and facilitates NRF2 ubiquitination and proteasomal degradation [2]. However, oxidants and electrophiles can interact with the numerous cystines in Keap1, causing conformational changes that disrupt the Keap1-NRF2 union releasing free NRF2. NRF2 may be phosphorylated by several exercise-stimulated kinases, such as extracellular signal-regulated kinases (ERK), protein kinase C (PKC), c-Jun N-terminal kinases (JNK), and p38 mitogen-activated protein kinases (p38 MAPK) [32,40,41]. NRF2 phosphorylation by these kinases prevents NRF2 degradation and facilitates its translocation to the nucleus and gene transcription [2]. The exercise-induced activation of the NRF2-regulated gene program plays a vital role in the adaptation to exercise training [39,42,43]. In contrast, serine phosphorylation of NRF2 by glycogen synthase kinase 3 beta (GSK3β) promotes its proteasomal degradation [44,45].

Although some antioxidants and XO inhibitors can partly block the acute signaling response to exercise [8,35], cell and animal experiments indicate that polyphenols such as mangiferin and quercetin may circumvent this drawback by inducing NRF2 [9,46,47]. However, no human study has determined whether dietary polyphenols may increase NRF2 levels and signaling in resting skeletal muscle. There is no information regarding polyphenols’ effects on the muscle signaling responses to exercise [8,48,49]. It also remains unknown whether Zynamite PX^®^ exerts signaling effects on resting skeletal muscle and whether short-term Zynamite PX^®^ supplementation modifies the signaling response to high-intensity exercise.

Therefore, this investigation aimed to determine the effects of 48 h Zynamite PX^®^ supplementation on skeletal muscle NRF2 protein levels and NRF2-induced signaling under basal conditions and in response to high-intensity exercise in humans. To achieve these aims, we have also determined the effects of Zynamite PX^®^ on Ca^2+^/calmodulin-dependent protein kinase II (CaMKII) and GSK3β and Keap1 as main regulatory mechanisms of NRF2 levels [2,50,51]. Thus, we hypothesized that Zynamite PX^®^ supplementation would increase basal NRF2 protein levels and attenuate the signaling responses induced by high-intensity exercise.

## 2. Materials and Methods

### 2.1. Subjects

Twenty-five young males agreed to take part in this research (means ± SD; age: 22.2 ± 2.1 years, body mass: 72.4 ± 7.4 kg, height: 177 ± 8 cm, body fat: 18.7 ± 5.1%, and maximal oxygen consumption (VO_2_max): 47.0 ± 6.4 mL kg^−1^ min^−1^) (Table 1). The criteria for eligibility included the following: (a) individuals aged between 18 and 35 years with a body mass index under 30 kg/m^2^, (b) gender: male, (c) a normal resting 12-lead electrocardiogram, and (d) an active lifestyle with regular exercise 2–4 times a week, though not necessarily adhering to a particular training regimen. The disqualifying criteria encompassed: (a) the presence of any illness or allergy, (b) any medical condition contraindicating physical activity, (c) habits such as smoking, and (d) undergoing any form of medical treatment. The study was carried out following approval by the Ethical Committee of the University of Las Palmas de Gran Canaria (CEIH-2015-03 and CEIH-2017-02). All volunteers were informed about the study’s aims and potential risks associated with the exercise and the invasive procedures and signed a written consent before starting the experiments. Participants were advised to avoid strenuous or unusual physical activities two days before all lab examinations and to abstain from beverages containing caffeine and alcohol the day preceding the preliminary tests and two days before the principal invasive experiment. In addition, they were instructed to continue with their usual dietary habits until the conclusion of the study. All the participants were physically fit and engaged in regular exercise.

### 2.2. General Overview

The research protocol was designed to identify the primary signaling routes triggered by cell stress during physical activity and post-exercise ischemia, utilizing a new experimental paradigm [36,52,53]. In this study, it was decided to allocate one subject to the Zynamite PX^®^ supplementation group per each 2 subjects included in the control group. It was determined that to identify a 40% difference in the basal NRF2 protein expression between the control group and the group that received supplements, a sample size of 7 and 15 participants was required, assuming α = 0.05 and β = 0.80, as calculated using G*Power version 3.1.9.6. Supplementation consisted of 140 mg of Zynamite^®^ (standardized to 60% mangiferin, an aqueous extract from *Mangifera indica*) [4] in combination with 140 mg of quercetin (provided as 280 mg *Sophora japonica* extract, standardized to 50% quercetin) every 8 h for two days (six doses in total). The control subjects did not receive any supplement. Supplemented subjects were informed that the study’s main aim was to examine the supplement’s effect on muscle signaling responses.

### 2.3. Pre-Tests and Familiarization

The first visit to the laboratory was dedicated to anthropometric measurements and assessing the body composition (dual-energy X-ray absorptiometry, Lunar iDXA, GE Healthcare, Milwaukee, WI, USA) [53]. Next, the participants reported to the laboratory on three other days for familiarization with experimental procedures, including an incremental exercise to exhaustion and sprint exercise (Wingate tests, a 30 s all-out sprint). This was continued by a session devoted to determining their VO_2_max using an incremental exercise to exhaustion [52] and another two sessions to measure their maximal functional reserve [52] using repeated supramaximal exercise bouts at 120% of VO_2_max until exhaustion, interspaced with 20 s recovery periods, one day recovering with a free circulation and the other with total occlusion of the circulation [52]. The exercise tests were carried out on a Lode ergometer (Groningen, The Netherlands), while subjects were requested to keep a pedaling frequency close to 80 revolutions per minute (RPMs) [52,53]. Exhaustion was defined by the subject abruptly ceasing to pedal or by a decrease in the pedaling cadence to less than 50 RPMs for 5 s despite intense verbal encouragement. The highest 20-s averaged VO_2_ value recorded during the incremental exercise to exhaustion or repeated supramaximal exercise bouts was taken as the VO_2_max [54]. Oxygen uptake was measured breath-by-breath using a metabolic cart (Vyntus, Jaeger-CareFusion, Höchberg, Germany) calibrated immediately before each test using high-grade certified gases provided by the manufacturer and validated by a butane combustion test [55]. The flowmeter was calibrated before each test at low (0.2 L/s) and high (2 L/s) flows.

### 2.4. Main Experiments and Supplement Administration

**Figure 1:**
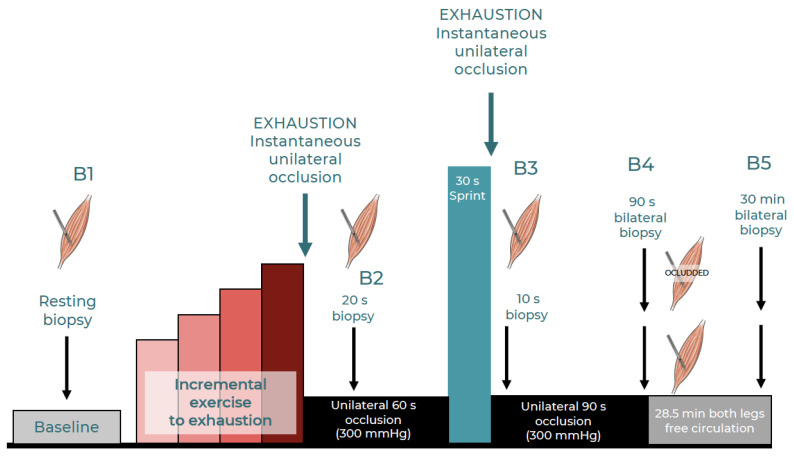
Schematic representation of the experimental protocol.

A schematic representation of the experimental protocol is presented in Figure 1. One week after the VO_2_max assessment, volunteers reported to the laboratory at 07:00 h, following a 12-h overnight fast. After resting supine for 90 min on a laboratory stretcher, the skin and subcutaneous tissue of the lateral aspect of the thigh was infiltrated with 1 mL of 2% lidocaine, and five min later, a baseline muscle biopsy was taken from one of the m. *vastus lateralis* (assigned randomly) using a Bergstrom’s biopsy needle with suction, as previously reported [53]. For the initial biopsy, the needle was angled distally at a 45° tilt [56], and the skin incision was covered with a temporary plaster that was easy to remove at exhaustion for fast biopsy collection.

Then, the contralateral thigh was also anesthetized in the same area by applying similar procedures, and a 5 mm incision was performed and covered with a temporary plaster. This was followed by the ingestion of the last dose of the supplement (140 mg of Zynamite^®^ with 140 mg of quercetin, marketed as Zynamite PX^®^). After that, an SC10D Hokanson cuff was wrapped around one of the thighs (chosen randomly, which was the thigh having an incision ready but not yet biopsied), as close as possible to the inguinal crease. The SC10D cuff was connected via a plastic tube to a fast cuff inflator (Hokanson, E20 AG101). Then, the subjects sat on the cycle ergometer. After verification of proper readings, the incremental exercise test started (~60 min after the ingestion of the supplement), with three minutes at 20 W, followed by 20 W increases every three minutes until the respiratory exchange ratio (RER) was ≥1.00. After that, the ergometer was unloaded while the subjects kept pedaling at 30–40 RPMs for two minutes. Then, the exercise intensity was raised to the same load attained at the end of the initial phase and incremented by 15 W every minute until exhaustion. Upon exhaustion, the Hokanson was triggered to apply a 300-mmHg pressure around the thigh to completely occlude the circulation in less than 2 s. Twenty seconds after exhaustion, the second muscle biopsy was taken from the occluded leg. This second biopsy was obtained by introducing the needle with a 45° inclination and pointing distally. Then, the volunteer remained quietly seated on the cycle ergometer and prepared to sprint maximally for 30 s (Wingate test) with the ergometer set in isokinetic mode (80 RPMs) exactly 60 s after exhaustion. During these 60 s, the cuff remained inflated, and the circulation occluded. At the beginning of the 30 s sprint, the cuff was instantaneously released and re-inflated at 300 mmHg at the end of the 30 s sprint. Exactly 10 s after the sprint, a third muscle biopsy was obtained from the occluded leg. During this third biopsy, the needle was inserted at a right angle to the thigh [56]. Immediately after the biopsy, the incision was covered, and the subject was moved carefully to a stretcher, where he rested in the supine position with the circulation of the leg biopsied fully occluded. Ninety seconds after the end of the exercise, a bilateral biopsy was simultaneously obtained from the occluded and non-occluded leg, immediately after which the cuff was deflated. In the occluded leg, the biopsy needle was introduced with a 45° inclination and pointing proximally, while in the contralateral leg, the needle was introduced perpendicular to the thigh. Thirty minutes after the end of the exercise, a last bilateral muscle biopsy was obtained. The needle was introduced with a 30° inclination and pointing proximally in the leg that had already undergone three biopsies. Thus, one leg had an occluded circulation of 60 s after the incremental exercise and another lasting 90 s after the sprint exercise, whilst the contralateral leg always recovered with free circulation. All biopsies were immediately frozen in liquid nitrogen and stored at −80 °C.

### 2.5. Muscle Protein Extraction and Western Blotting

Skeletal muscle lysates were obtained by grinding 10 mg of muscle sample for one minute using a Mikro-Dismembrator S (Sartorius, Goettingen, Germany) equipped with stainless steel balls. The ground muscle was immediately homogenized in urea lysis buffer containing 6 M urea and 1% SDS, along with 10X PhosSTOP phosphatase inhibitor (Cat. #4906837001) and 50X Complete protease inhibitor (Cat. #11697498001) obtained from Roche (Basel, Switzerland). Subsequently, the muscle lysate was centrifuged (25,200× *g* for 12 min at 16 °C). Total protein content was quantified using the bicinchoninic acid assay [57]. The extract volume was adjusted to the muscle weighted individually to achieve a ~6.8 μg/μL final concentration in all muscle protein extracts. Afterwards, the supernatant was combined with an electrophoresis loading buffer. This buffer consisted of 160 mM Tris-HCl at 6.8 pH, 5.9% SDS, 25.5% glycerol, along with 15% β-mercaptoethanol-bromophenol blue.

The amount of protein to be loaded and the antibody concentration for each assay was determined empirically by loading different amounts of control protein (2 to 30 μg). The control protein was prepared using human skeletal muscle processed with the same procedures applied to the experimental samples. The range of protein amount for which there was a linear relationship between the amount of control protein and optical band density was determined for each antibody. Later, an amount of sample protein within the linear range of the protein/optical density response was loaded for each kinase or signaling protein tested (7.5 to 15 μg), followed by electrophoresis on SDS-PAGE gels by the Laemmli system. The proteins were then transferred onto Immun-Blot polyvinylidene fluoride (PVDF) membranes for immunoblotting (Bio-Rad Laboratories, Hercules, CA, USA) (Supplementary Table S1). Loading and transfer efficiency was assessed by staining the membranes with Reactive Brown 10 (Sigma Aldrich, St. Louis, MO, USA). All samples corresponding to a given participant were run on the same gel intercalated with four control samples.

The membranes underwent a blocking process for a span of one hour using either 5% non-fat dried milk powder diluted in Tris-buffered saline containing 0.1% Tween 20 (Blotto) or 4% bovine serum albumin (BSA). This was followed by an overnight incubation period of 12–15 h at a temperature of 4 °C in the presence of primary antibodies. These primary antibodies were diluted in 5% Blotto or 4% BSA blocking buffers. Right after, the membranes were exposed to an HRP-linked anti-rabbit or anti-mouse antibody, the dilution of which ranged from 1:5000 to 1:20,000 in 5% Blotto-blocking buffer in every case. The membranes were then subjected to chemiluminescent visualization using the Clarity™ Western ECL Substrate procured from Bio-Rad Laboratories (Hemel Hempstead, UK) through a ChemiDocTM Touch Imaging System also from Bio-Rad Laboratories (Hercules, CA, USA). Lastly, the densitometric band data were quantified employing the Image Lab^©^ software, version 6.0.1 from Bio-Rad Laboratories. The densitometric measurements were expressed in arbitrary units (a.u). No further corrections were carried out because the loading was consistent across membranes. Each sample was assessed with a single measurement taken per sample. The supplemented group had three missing values: the biopsy corresponding to the post-sprint exercise in one subject and the two corresponding to the 90 min post exercise in another subject.

The antibodies employed in the current study were acquired from different suppliers. From Abcam (Cambridge, MA, USA): Ser^40^-NRF2 (no. ab76026) and total NRF2 (no. ab62352). From Cell Signaling Technology (Danvers, MA, USA): Thr^287^-CaMKII (no. 12716), Thr^180^/Tyr^182^-p38 MAPK (no. 9211), Ser^9^-GSK3β (no. 5558) and Catalase (no. 14097). From Proteintech (Rosemont, IL, USA): Glutathione reductase (GR) (no. 18257-1-AP) and Keap1 (no. 10503-2-AP). The horseradish peroxidase-conjugated goat anti-rabbit secondary antibody (product no. 111-035-144), and the horseradish peroxidase-conjugated goat anti-mouse antibody (product no. 115-035-003) were both ordered from Jackson ImmunoResearch (West Grove, PA, USA). The CaMKIIδ_D_ isoform was identified by using an isoform-specific antibody for CaMKIIδ_D_ (anti-CaMKII delta isoform no. A010-55AP; Badrilla, Leeds, UK) [58,59]. See Supplementary Table S1 for more details regarding the antibodies and specific procedures. The Protein Plus Precision All Blue Standards were supplied by Bio-Rad Laboratories (Hercules, CA, USA).

### 2.6. Statistical Analysis

The values are reported as means ± standard deviations. The hypothesis of normality for each variable was assessed via the Shapiro–Wilks test. A logarithmic transformation was applied for variables demonstrating a significant departure from the Gaussian distribution. Unpaired *t*-tests were used to compare the mean expression values under basal conditions between the supplemented and the control group. A paired *t*-test was used to determine whether there was a difference between the leg that recovered with ischemia and the leg recovering with free circulation at 90 s and 30 min. Since both legs had similar responses at the 90 s biopsy, these two biopsies were averaged, and the resulting value was taken as representative of the 90 s post-exercise response. A similar approach was used for the 30 min responses since non-significant differences were observed between legs at this time point. Then, a repeated-measures ANOVA was run with one within-subjects factor: exercise (with five levels: resting (T1), post-VO_2_max (T2), 10 s post-Wingate test (T3), 90 s post-Wingate test (T4), and 30 min post-Wingate test (T5)) and supplementation as between-subjects factors. The pre-requisite assumption of sphericity was verified using Mauchly’s test of sphericity prior to the execution of the ANOVA. When the assumption of sphericity was violated, the degrees of freedom (*df*) were corrected using the Huynh–Feldt epsilon procedure. The alpha level of statistical significance was established at *p* ≤ 0.05. The statistical analyses were undertaken with IBM SPSS software, version 29.0, designed specifically for Apple Computers (IBM, New York, NY, USA).

## 3. Results

### 3.1. Effects of Zynamite PX^®^ Supplementation on Basal Signalling

**Figure 2:**
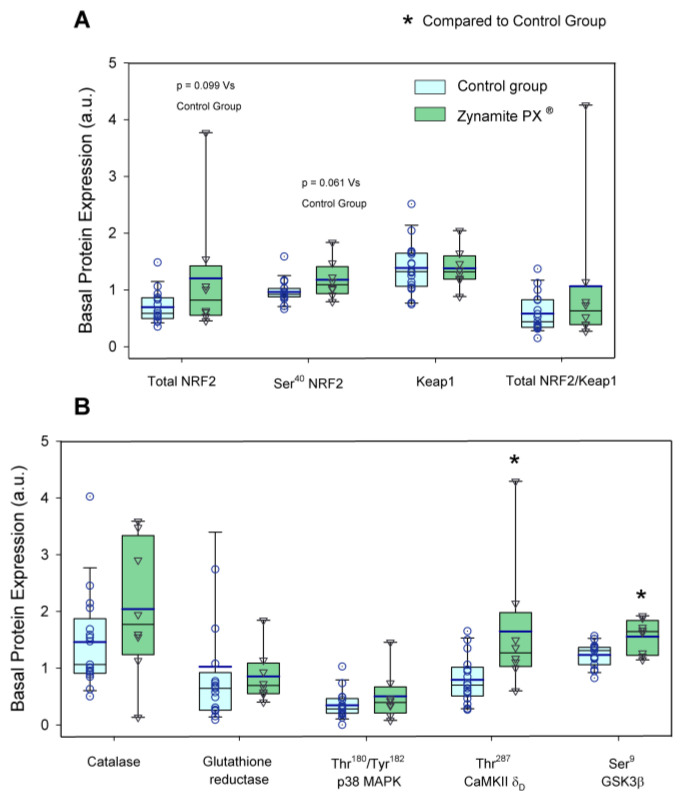
Protein expression levels of the enzymes and transcription factors measured under basal conditions. (**A**) Values for basal total NRF2, Ser^40^-NRF2, Keap1, and total NRF2/Keap1 ratio (a.u.) and (**B**) values for catalase, glutathione reductase, Thr^180^/Tyr^182^-p38 MAPK, Thr^287^-CaMKIIδ_D_, and Ser^9^-GSK3β (a.u.). The whiskers delimit the 5th and 95th percentiles; the thin and thick horizontal lines correspond to the median and the mean values, respectively; and the upper and lower ends of the boxes define the 1st and 3rd quartiles, respectively. Control group: circles (*n* = 17) and Zynamite PX^®^ group triangles (*n* = 8). * *p* < 0.05 compared to the control group.

**Figure 3:**
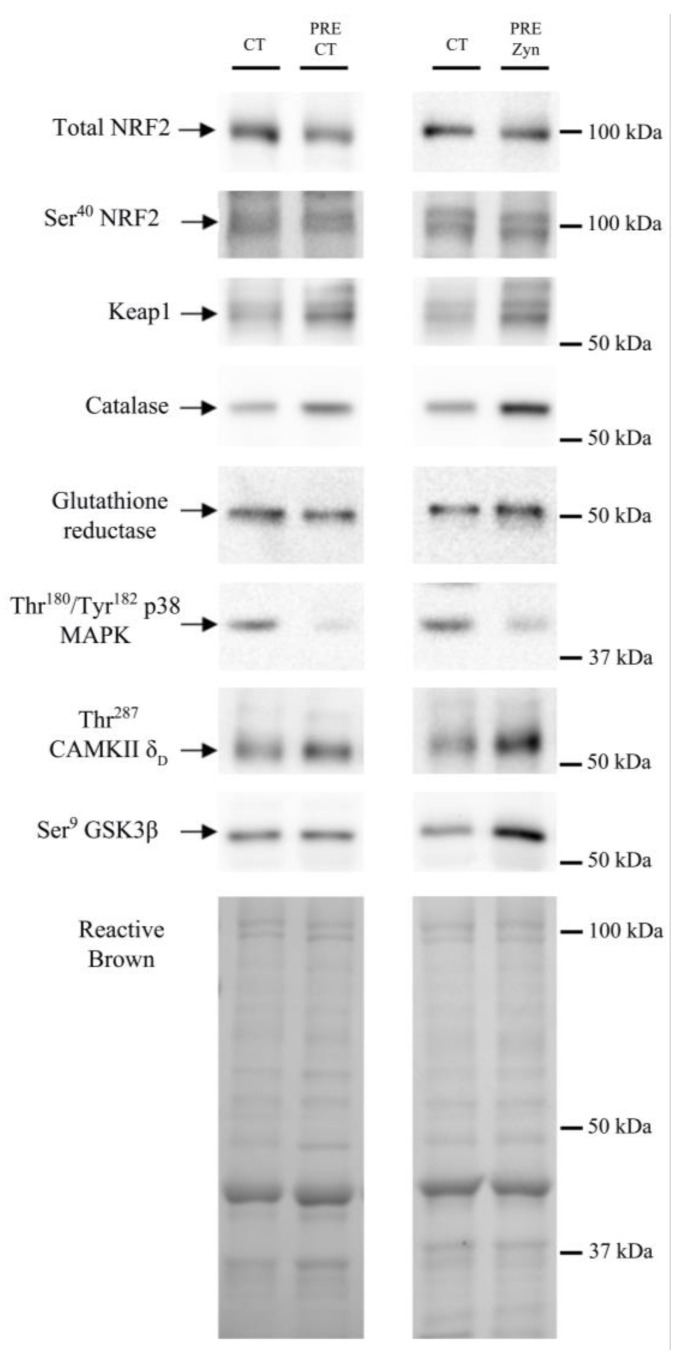
Immunoblots and total amount of protein loaded (Reactive Brown Staining) from a representative subject of the control group (Pre CT) and the Zynamite PX^®^ supplemented group (Pre Zyn). CT corresponds to a human control sample (non-experimental) loaded onto each gel to allow for normalization and loading control. The markers indicate the closest molecular weight in kDa.

The protein expression levels of the enzymes and transcription factors measured under basal conditions are depicted in Figure 2 and Figure 3. Basal total NRF2 and Ser^40^-NRF2 protein expression were 1.7- and 1.2-fold higher in the Zynamite PX^®^-supplemented than in the control group (*p* = 0.099 and *p* = 0.061, respectively) (Figure 2A). Both groups had similar basal levels of Keap1 (*p* = 0.975) and total NRF2/Keap1 ratio (*p* = 0.247) (Figure 2A). The basal protein expression levels of catalase and glutathione reductase were similar in both groups (*p* = 0.596 and *p* = 0.481, respectively) (Figure 2B). Thr^180^/Tyr^182^-p38 MAPK protein expression was similar at pre in both groups (*p* = 0.252) (Figure 2B). Thr^287^-CaMKIIδ_D_ basal expression was 2.1-fold higher in the Zynamite PX^®^ supplemented than in the control group (*p* = 0.007) (Figure 2B). This was accompanied by 1.3-fold higher basal Ser^9^-GSK3β phosphorylation in the Zynamite PX^®^ supplemented group than in the control group (*p* = 0.005) (Figure 2B).

### 3.2. Effects of Zynamite PX^®^ Supplementation Signalling Response to Exercise

**Figure 4:**
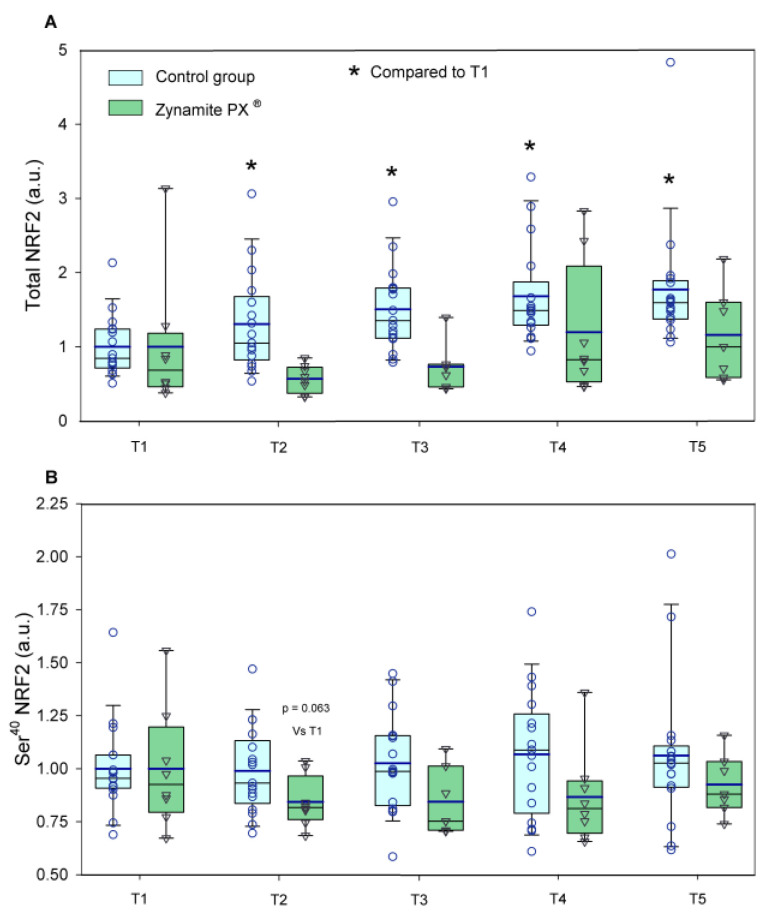
Protein expression levels of the enzymes and transcription factors during the exercise conditions and recovery. (**A**) Values for total NRF2 and (**B**) Ser^40^-NRF2 (a.u.). Baseline (T1), post-VO_2_max (T2), 10 s post-Wingate test (T3), 90 s post-Wingate test (T4), and 30 min post-Wingate test (T5). The whiskers delimit the 5th and 95th percentiles; the thin and thick horizontal lines correspond to the median and the mean values, respectively; and the upper and lower ends of the boxes define the 1st and 3rd quartiles, respectively. Control group: circles (*n* = 17) and Zynamite PX^®^ group triangles (*n* = 8). * *p <* 0.05 compared to T1. Total NRF2: ANOVA exercise effect *p <* 0.001; exercise × supplementation interaction *p =* 0.032; Ser^40^-NRF2: ANOVA exercise effect *p =* 0.632; exercise × supplementation interaction *p =* 0.597.

**Figure 5:**
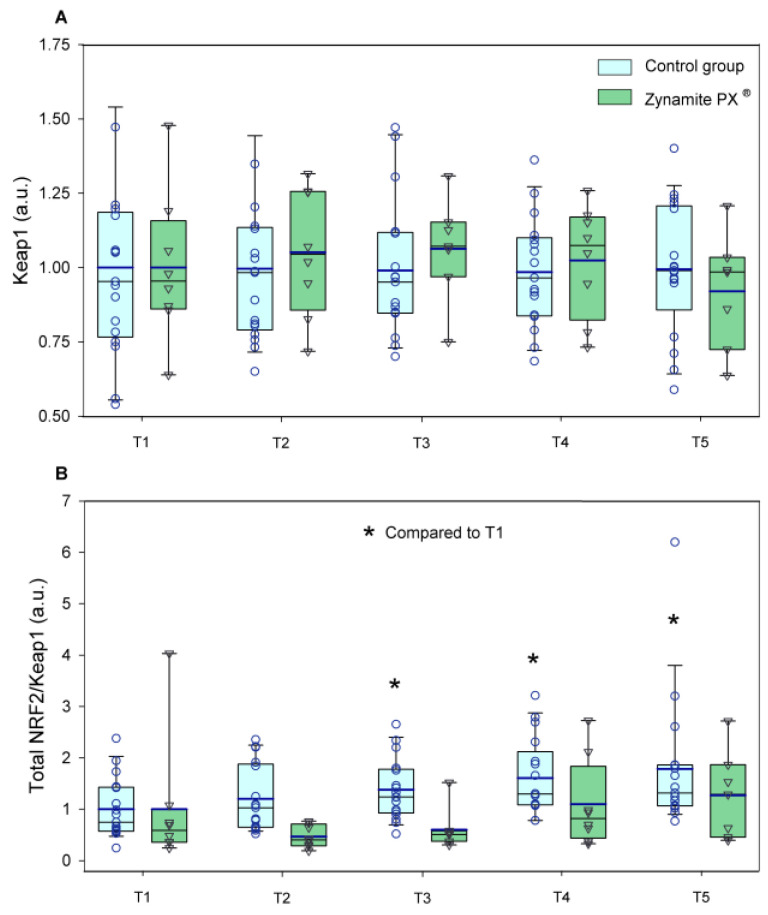
Protein expression levels of the enzymes and transcription factors during the exercise conditions and recovery. (**A**) Values for Keap1, and (**B**) total NRF2/Keap1 ratio (a.u.). Baseline (T1), post-VO_2_max (T2), 10 s post-Wingate test (T3), 90 s post-Wingate test (T4), and 30 min post-Wingate test (T5). The whiskers delimit the 5th and 95th percentiles; the thin and thick horizontal lines correspond to the median and the mean values, respectively; and the upper and lower ends of the boxes define the 1st and 3rd quartiles, respectively. Control group: circles (*n* = 17) and Zynamite PX^®^ group triangles (*n* = 8). * *p <* 0.05 compared to T1. Keap1: ANOVA exercise effect *p =* 0.925; exercise × supplementation interaction *p =* 0.921; NRF2/Keap1: ANOVA exercise effect *p <* 0.001; exercise × supplementation interaction *p =* 0.061.

**Figure 6:**
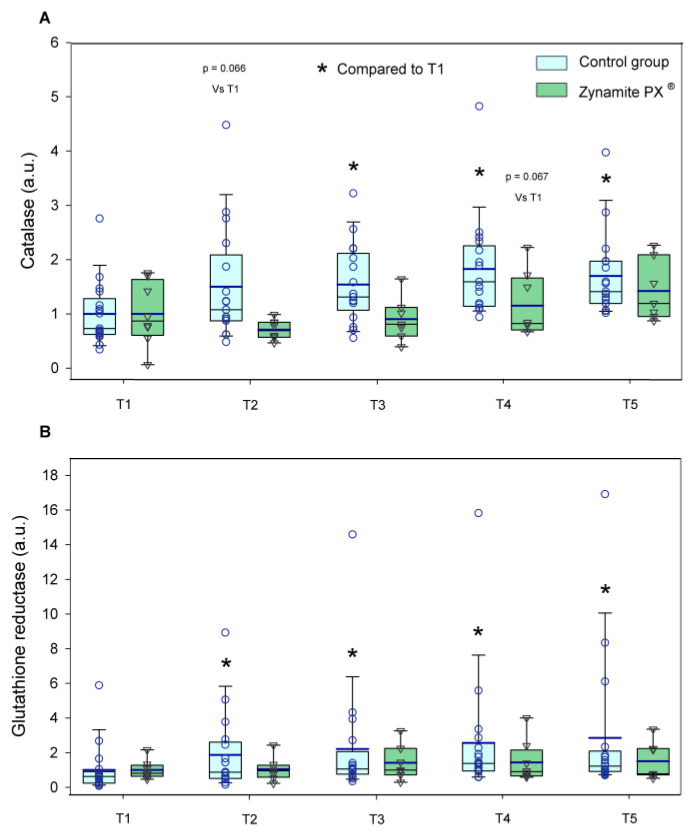
Protein expression levels of the enzymes and transcription factors during the exercise conditions and recovery. (**A**) Values for catalase, and (**B**) glutathione reductase (a.u.). Baseline (T1), post-VO_2_max (T2), 10 s post-Wingate test (T3), 90 s post-Wingate test (T4), and 30 min post-Wingate test (T5). The whiskers delimit the 5th and 95th percentiles; the thin and thick horizontal lines correspond to the median and the mean values, respectively; and the upper and lower ends of the boxes define the 1st and 3rd quartiles, respectively. Control group: circles (*n* = 17) and Zynamite PX^®^ group triangles (*n* = 8). * *p <* 0.05 compared to T1. Catalase: ANOVA exercise effect *p =* 0.003; exercise × supplementation interaction *p =* 0.033; Glutathione reductase: ANOVA exercise effect *p =* 0.004; exercise × supplementation interaction *p* = 0.130.

Total NRF2 expression was increased by exercise only in the control group (1.3–1.7-fold, ANOVA exercise effect *p <* 0.001, F = 8.09, *df* = 4; exercise ×supplementation interaction *p =* 0.032, F = 2.79, *df* = 4) (Figure 4A). Ser^40^-NRF2 protein expression remained unchanged with exercise in both groups (ANOVA exercise effect *p =* 0.632; F = 0.60, *df* = 3.3; exercise × supplementation interaction *p =* 0.597, F = 0.66, *df* = 3.3) (Figure 4B). Although non-significant changes were observed in Keap1 with exercise in either group (ANOVA exercise effect *p =* 0.925, F = 0.22, *df* = 4; exercise × supplementation interaction *p =* 0.921, F = 0.23, *df* = 4) (Figure 5A), the total NRF2/Keap1 ratio was increased by exercise only in the control group (1.2–1.8-fold, ANOVA exercise effect *p <* 0.001, F = 6.94, *df* = 4; exercise × supplementation interaction *p =* 0.061, F = 2.35, *df* = 4) (Figure 5B). Catalase protein expression was increased in response to exercise only in the control group (1.5–1.8-fold, ANOVA exercise effect *p =* 0.003, F = 4.32, *df* = 4; exercise × supplementation interaction *p =* 0.033, F = 2.75, *df* = 4) (Figure 6A). Glutathione reductase protein expression was increased in response to exercise in the control group (1.9–2.9-fold, ANOVA exercise effect *p =* 0.004, F = 4.12, *df* = 4; exercise × supplementation interaction *p =* 0.130, F = 1.84, *df* = 4) (Figure 6B).

**Figure 7:**
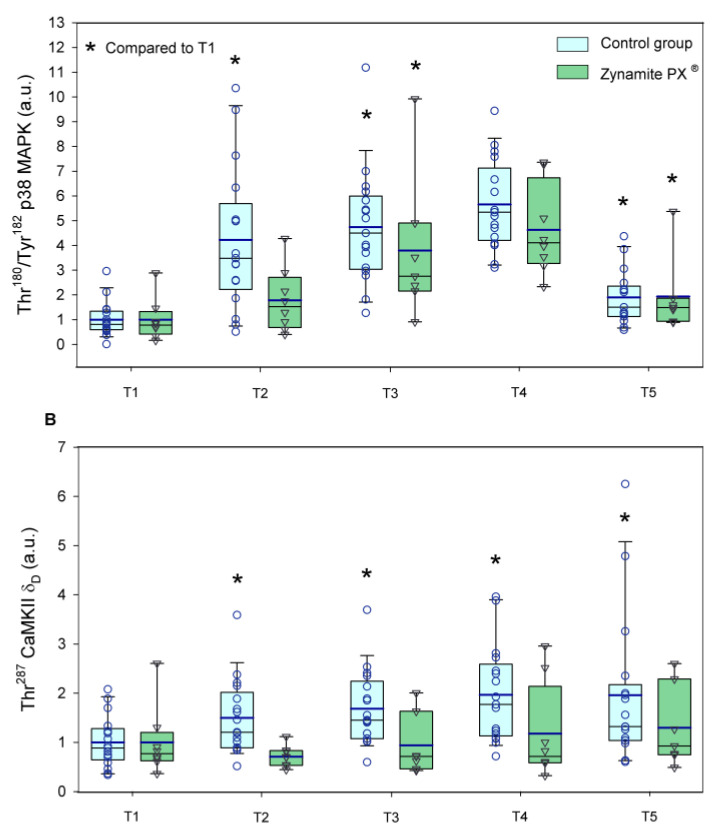
Protein expression levels of the enzymes and transcription factors during the exercise conditions and recovery. (**A**) Values for Thr^180^/Tyr^182^-p38 MAPK, and (**B**) Thr^287^-CaMKIIδ_D_ (a.u.). Baseline (T1), post-VO_2_max (T2), 10 s post-Wingate test (T3), 90 s post-Wingate test (T4), and 30 min post-Wingate test (T5). The whiskers delimit the 5th and 95th percentiles; the thin and thick horizontal lines correspond to the median and the mean values, respectively; and the upper and lower ends of the boxes define the 1st and 3rd quartiles, respectively. Control group: circles (*n* = 17) and Zynamite PX^®^ group triangles (*n* = 8). * *p <* 0.05 compared to T1. Thr^180^/Tyr^182^-p38 MAPK: ANOVA exercise effect *p <* 0.001; exercise × supplementation interaction *p* = 0.080; Thr^287^-CaMKIIδ_D_: ANOVA exercise effect *p* = 0.008; exercise × supplementation interaction *p* = 0.064.

**Figure 8:**
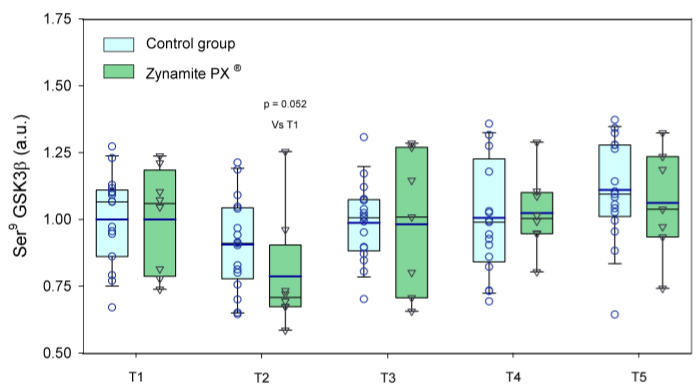
Protein expression levels of Ser^9^-GSK3β (a.u.) during the exercise conditions and recovery. Baseline (T1), post-VO_2_max (T2), 10 s post-Wingate test (T3), 90 s post-Wingate test (T4), and 30 min post-Wingate test (T5). The whiskers delimit the 5th and 95th percentiles; the thin and thick horizontal lines correspond to the median and the mean values, respectively; and the upper and lower ends of the boxes define the 1st and 3rd quartiles, respectively. Control group: circles (*n* = 17) and Zynamite PX^®^ group triangles (*n* = 8). ANOVA exercise effect *p <* 0.001; exercise × supplementation interaction *p =* 0.256.

**Figure 9:**
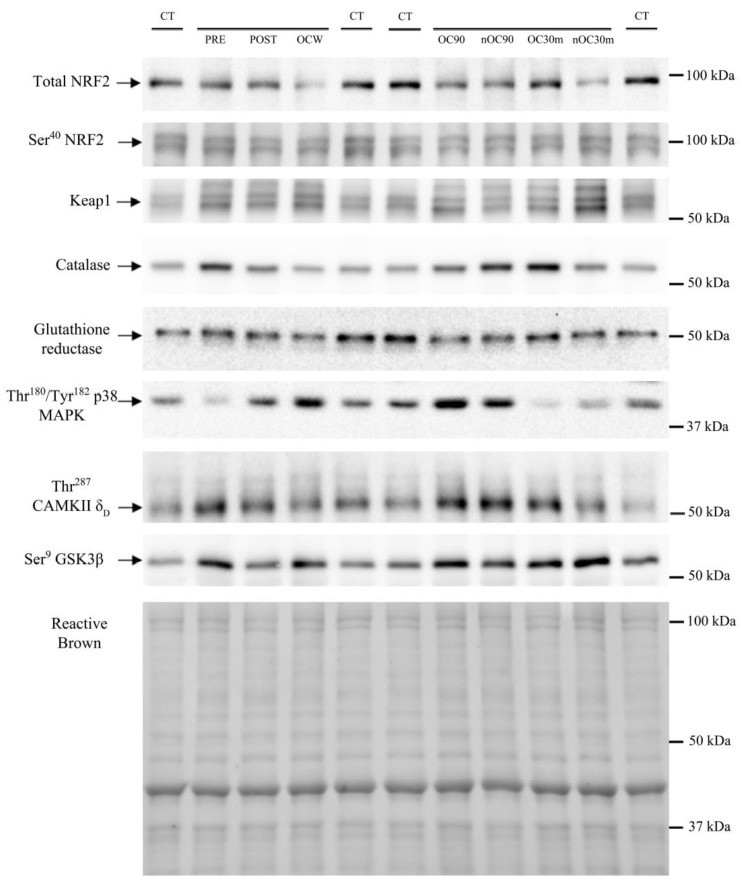
Immunoblots and total amount of protein loaded (Reactive Brown Staining) from a representative subject of the Zynamite PX^®^ supplemented group. Images from top to bottom: total NRF2, Ser^40^-NRF2, Keap1, catalase, glutathione reductase, Thr^180^/Tyr^182^-p38 MAPK, Thr^287^-CaMKIIδ_D_, and Ser^9^-GSK3β. CT corresponds to a human control sample (non-experimental) loaded onto each gel in quadruplicate as a loading control. An illustration of experimental phases is depicted in Figure 1. PRE, before exercise. POST, 20 s after the end of the incremental exercise with ischemic recovery from the occluded leg. OCW, OC90, and OC30m, 10 s, 90 s, and 30 min after the end of the sprint, respectively, all from the occluded leg. nOC90 and nOC30m, 90 s and 30 min, respectively, from the leg recovering with free circulation. The markers indicate the closest molecular weight in kDa.

**Figure 10:**
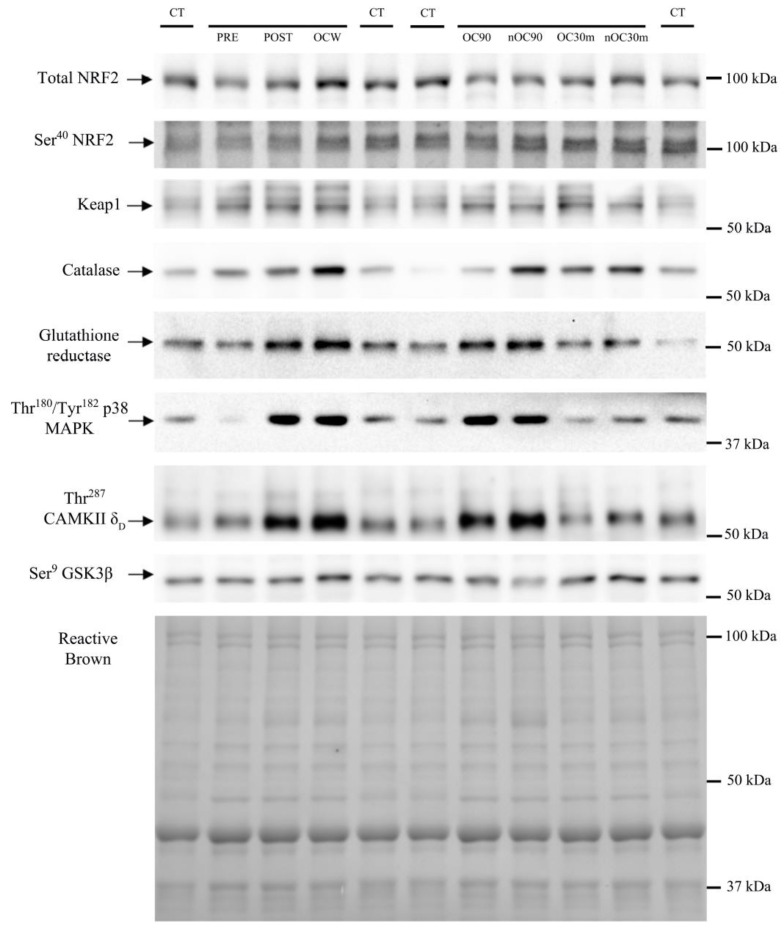
Immunoblots and total amount of protein loaded (Reactive Brown Staining) from a representative subject of the control group. Images from top to bottom: total NRF2, Ser^40^-NRF2, Keap1, catalase, glutathione reductase, Thr^180^/Tyr^182^-p38 MAPK, Thr^287^-CaMKIIδD, and Ser^9^-GSK3β. CT corresponds to a human control sample (non-experimental) loaded onto each gel in quadruplicate as a loading control. An illustration of experimental phases is depicted in Figure 1. PRE, before exercise. POST, 20 s after the end of the incremental exercise with ischemic recovery from the occluded leg. OCW, OC90, and OC30m, 10 s, 90 s, and 30 min after the end of the sprint, respectively, all from the occluded leg. nOC90 and nOC30m, 90 s, and 30 min, respectively, from the leg recovering with free circulation. The markers indicate the closest molecular weight in kDa.

Thr^180^/Tyr^182^-p38 MAPK protein expression was similarly elevated in response to exercise in both groups (1.8–5.7-fold, ANOVA exercise effect *p* < 0.001, F = 35.3, *df* = 2.51; exercise × supplementation interaction *p =* 0.080, F = 2.49, *df* = 2.51) (Figure 7A). Thr^180^/Tyr^182^-p38 MAPK protein expression was slightly higher in the leg recovering with ischemia (*p =* 0.01, F = 7.89, *df* = 1); however, there was no significant interaction for the comparison of the responses observed in the leg with ischemia and the leg with free circulation recovery regardless of supplementation (recovery time × ischemia interaction *p =* 0.845, F = 0.03, *df* = 1; recovery time × ischemia × supplementation interaction *p =* 0.830, F = 0.05, *df* = 1). Thr^287^-CaMKIIδ_D_ protein expression was increased in response to exercise in the control group (1.5–2.0-fold, ANOVA exercise effect *p =* 0.008, F = 3.72, *df* = 4; exercise × supplementation interaction *p =* 0.064, F = 2.31, *df* = 4) (Figure 7B). Thr^287^-CaMKIIδ_D_ protein expression was slightly higher in the leg recovering with ischemia (*p =* 0.033, F = 5.19, *df* = 1); nevertheless, there was no significant interaction for the comparison of the responses observed in the leg with ischemia and the leg with free circulation recovery regardless of supplementation (recovery time × ischemia interaction *p =* 0.403, F = 0.73, *df* = 1; recovery time × ischemia × supplementation interaction *p =* 0.628, F = 2.41, *df* = 1). Ser^9^-GSK3β phosphorylation was reduced by 13% after the incremental exercise to exhaustion and then recovered pre-exercise values in both groups (ANOVA exercise effect *p* < 0.001, F = 5.79, *df* = 4; exercise × supplementation interaction *p =* 0.256, F = 1.36, *df* = 1) (Figure 8). Representative immunoblots of the enzymes and protein assessed during exercise can be seen in Figure 9 and Figure 10.

## 4. Discussion

**Figure 11:**
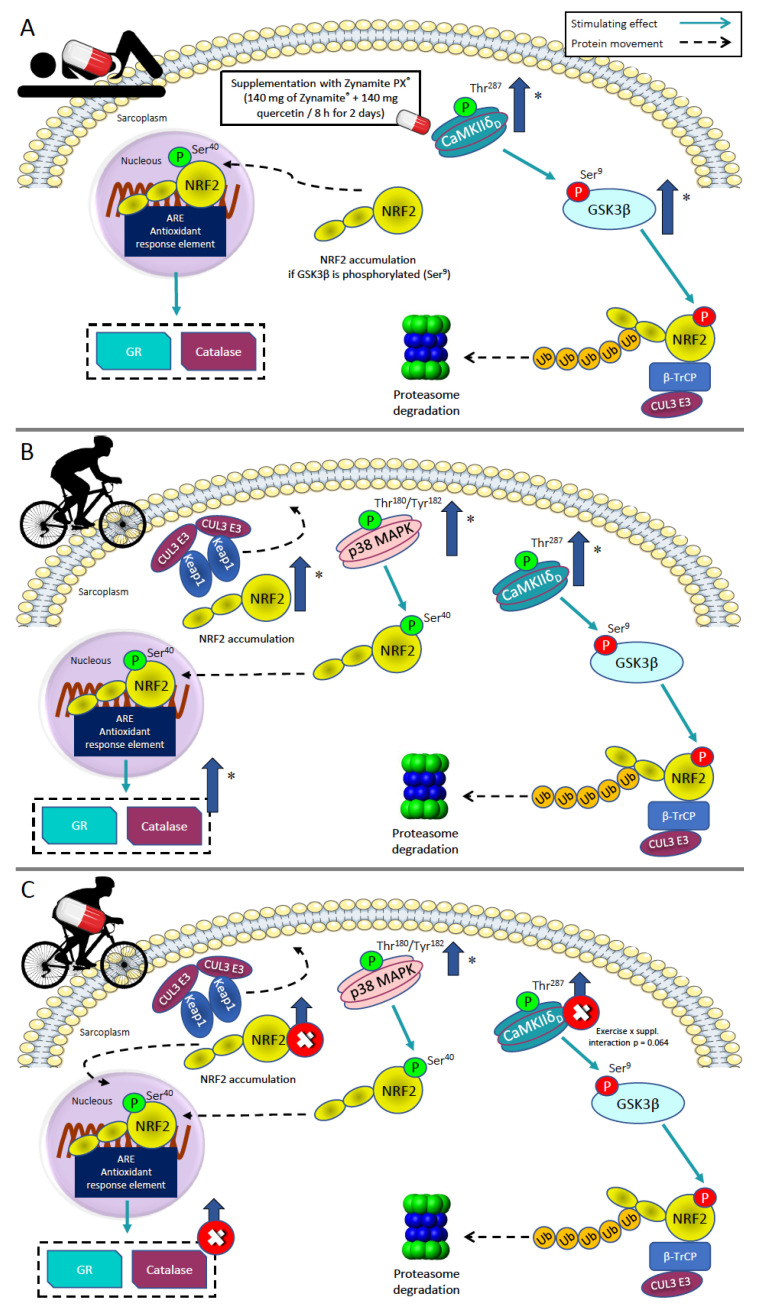
Schematic models of the NRF2 signaling pathway and its regulatory mechanisms in human skeletal muscle. (**A**) Basal signaling after 48 h of Zynamite PX^®^ supplementation. * *p* < 0.05 compared to the control group. (**B**) Signaling elicited by high-intensity exercise without supplementation. * *p <* 0.05 compared to T1. (**C**) Signaling elicited by high-intensity exercise after supplementation with Zynamite PX^®^. * *p <* 0.05 compared to T1. Stimulating effects are represented by thin blue connecting lines. Changes in cellular localizations are presented with black dashed lines. The thick arrows in darker blue color located close to the specific markers illustrate this study’s overall protein expression changes. Red crosses indicate suppression of the expected signaling response to high-intensity exercise following supplementation with Zynamite PX^®^. Activatory/inhibitory phosphorylations are depicted in green/red circles.

The present investigation has shown that the oral administration of Zynamite PX^®^, a blend of natural polyphenols combining an extract from mango leaves abundant in mangiferin with a small amount of quercetin, elicits significant changes in resting skeletal muscle signaling molecules and modifies the signaling responses to high-intensity exercise (Figure 11). Our volunteers ingested 140 mg of mango leaf extract combined with 140 mg of quercetin every eight hours for a total of six doses before the resting biopsy. This was associated with increased CaMKII phosphorylation (Thr^287^-CaMKIIδ_D_), resulting in a stimulation of the activity of the enzyme, which phosphorylates and inhibits GSK3β [51] in the muscle biopsies obtained at rest. In unstressed conditions, GSK3β is constitutively active and phosphorylates NRF2 in C-terminal residues (different from Ser^40^). This phosphorylation facilitates the interaction of NRF2 with β-transducin repeat-containing E3 ubiquitin-protein ligase (β-TrCP), leading to NRF2 ubiquitination and subsequent proteasomal degradation [50]. Thus, our results indicate that Zynamite PX^®^ elicits the phosphorylation and inhibition of GSK3β, facilitating an increase in resting levels of NRF2 in human skeletal muscle [50,60]. This interpretation is further supported by the observed 1.7-fold higher levels of NRF2 in the resting biopsies of the supplemented participants, which despite not achieving statistical significance, should not be ignored. Moreover, the level of Ser^40^-NRF2 phosphorylation showed a similar trend, i.e., it was slightly increased in the supplemented participants. Ser^40^-NRF2 phosphorylation prevents NRF2 degradation and facilitates its translocation to the nucleus and gene transcription [2]. However, no significant changes were observed in the basal protein expression levels of catalase and glutathione reductase, whose genes are regulated by NRF2. Thus, a more prolonged intake of Zynamite PX^®^ may be required to upregulate the expression of these two genes. Nevertheless, at least in the case of catalase, this enzyme is tightly regulated, being acutely increased under conditions of increased ROS production, for example, high-intensity exercise, but immediately downregulated at the end of exercise [36].

### 4.1. Potential Benefits Associated with Increased Inhibition of GSK3β

The present investigation shows that Zynamite PX^®^ oral intake increases Ser^9^-GSK3β phosphorylation, a mechanism that reduces its enzymatic activity [61,62]. Exercise also elicits Ser^9^-GSK3β phosphorylation [63,64]. Increased Ser^9^-GSK3β phosphorylation facilitates the preservation of muscle mass, protecting against muscle wasting due to disuse, chronic diseases, or caloric restriction [65,66,67,68], and facilitates myogenic differentiation and myoblast fusion [69,70]. Inactivation of GSK3β is necessary for the activation of muscle glycogen synthase [71,72], which is responsible for muscle glycogen resynthesis after exercise [73].

### 4.2. Zynamite PX^®^ Increases Basal Levels of CaMKII Phosphorylation (Thr^287^-CaMKIIδ_D_)

Ca^2+^/calmodulin-dependent protein kinase II (CaMKII) plays a critical role in the regulation of muscle metabolism [74,75,76], muscle intracellular pH [77], calcium homeostasis [78], mitochondrial function [79] and biogenesis [80,81], redox balance [82], insulin sensitivity [83,84], and muscle growth [85], being strongly activated in response to exercise [86]. Increased CaMKIIδ_D_ expression and phosphorylation have been reported in skeletal muscle under basal conditions after sprint training [87] and strength training [59]. Interestingly, an association has been reported between the increase in Thr^287^-CaMKIIδ_D_ phosphorylation and the magnitude of muscle hypertrophy elicited by a strength training program in humans [59].

### 4.3. Zynamite PX^®^ Supplementation Attenuates the Activation of Stress Kinases and Redox Signalling in Response to High-Intensity Exercise to Exhaustion

This investigation shows that after brief supplementation with Zynamite PX^®^, the unstressed skeletal muscle displays some molecular adaptations like those elicited by exercise. Usually, exercise elicits Thr^287^-CaMKII and Thr^180^/Tyr^182^-p38 MAPK phosphorylation [32,40,88], as observed in the present investigation in the control group. Thr^287^-CaMKII phosphorylation is more prominent after sprint exercise [35] and workouts eliciting more metabolite accumulation [32], but it is also observed after prolonged exercise [89]. Nevertheless, when the exercise intensity is lower or elicits lesser metabolic stress, as observed in the trained state [90], the Thr^287^-CaMKII phosphorylation response to exercise is attenuated or blunted [32,89]. The present investigation demonstrates that Zynamite PX^®^ supplementation attenuates the expected Thr^287^-CaMKII and Thr^180^/Tyr^182^-p38 MAPK phosphorylation observed in the control group. At least three mechanisms could explain this effect. Firstly, the exercise-mimetic action elicited by the 48 h Zynamite PX^®^ supplementation may have attenuated the signaling response to exercise, as usually observed after training in humans [90]. Secondly, the marked increased basal phosphorylation of GSK3β after Zynamite PX^®^ supplementation might have facilitated some NRF2 activity prior to the exercise resulting in lower ROS-induced signaling due to either lower ROS production or enhanced quenching during the exercise. In agreement, total NRF2 expression and the NRF2/Keap1 ratio were increased by exercise only in the control group, probably because NRF2 was already elevated before the start of exercise in the Zynamite PX^®^ supplemented group. This explanation is supported by the fact that catalase and glutathione reductase, the antioxidant enzymes whose gene expression is stimulated by NRF2, were increased in response to exercise only in the control group. Thirdly, the dose of Zynamite PX^®^ administered 60 min before the start of exercise may have contributed to abrogating part of the ROS signaling response in a similar way as observed after the ingestion of antioxidant cocktails [35] or inhibitors of XO [91]. Although it has been suggested that antioxidant ingestion before exercise may blunt part of the adaptations to exercise, this does not seem to be the case in healthy humans [92] and may be observed only after the intake of high doses of vitamin C and E [93,94]. In turn, the intake of fruit-derived polyphenols is considered favorable to enhance performance and recovery in athletes [1].

### 4.4. Limitations

This investigation has several limitations. First, some of the reported effects had “*p*” values between 0.05 and 0.1 for some of these effects; therefore, we cannot exclude the possibility of a type II error for some comparisons. Second, the changes reported in basal protein expression levels were observed ten hours after administering the last of the six Zynamite PX^®^ administered. Whether more marked changes would be seen after a more prolonged supplementation remains unknown. Third, it is yet to be determined which of the polyphenols included in Zynamite PX^®^ contributes the most to the reported effects or if they are partly due to specific metabolites generated by the gut microbiota entering the circulation. Fourth, only males were tested in the present investigation; nevertheless, we have previously shown that Zynamite PX^®^ improves performance and enhances recovery in males and females [4,6,95].

## 5. Conclusions

The oral intake of Zynamite PX^®^ increases basal Thr^287^-CaMKIIδ_D_ and GSK3β phosphorylation in human skeletal muscle, which may elicit muscle adaptations to some extent, like those elicited by exercise. Consequently, the stress kinases’ responses to exercise are partly blunted after Zynamite PX^®^ supplementation. The increase in GSK3β phosphorylation may be associated with additional benefits which have not been assessed in the present investigation.

Future studies should determine whether a more prolonged supplementation may be associated with additional beneficial outcomes in males and females. GSK3β inhibits glycogen synthase; therefore, future studies should determine whether Zynamite PX^®^ could accelerate muscle glycogen synthesis by inhibiting GSK3β and facilitate post-exercise recovery. Another aspect worth studying is the potential effects of Zynamite PX^®^ on muscle protein synthesis, which the inhibition of GSK3β may facilitate [67].

## Figures and Tables

**Table 1:** Physical characteristics, body composition, and VO_2_max.

	Control Group (*n* = 17)	Zynamite PX^®^ Group (*n* = 8)	*p*
Age (years)	22.5 ± 2.4	21.6 ± 1.2	0.350
Height (cm)	178 ± 8	177 ± 9	0.786
Weight (kg)	72.7 ± 7.6	71.6 ± 7.4	0.730
Body fat (%)	18.6 ± 5.8	18.8 ± 3.6	0.930
Fat body mass (kg)	13.7 ± 5.3	13.6 ± 3.7	0.930
Lean body mass (kg)	55.8 ± 5.1	54.9 ± 5.1	0.698
VO_2_max (mL min^−1^)	3432 ± 489	3281 ± 367	0.448
VO_2_max (mL kg^−1^ min^−1^)	47.5 ± 7.1	46.0 ± 4.7	0.594

## Data Availability

Deidentified participant data are available from the senior author on reasonable request for research purposes.

## Supplementary Materials

The following supporting information can be downloaded at: https://www.mdpi.com/article/10.3390/nu15132848/s1, Table S1: Detailed description of Western blotting antibodies and procedures.
