# Supplementary material for: A Mango Leaf Extract (Zynamite®) Combined with Quercetin Has Exercise-Mimetic Properties in Human Skeletal Muscle
Source: Nutrients. 2023 Jun 23;15(13):2848. doi: 10.3390/nu15132848 (PMC10346248; doi:10.3390/nu15132848)
Supplement: Supplementary file 1 [file nutrients-15-02848-s001.zip › nutrients-2464739-supplementary.pdf]

Supplementary Table S1. Detailed description of Western blotting antibodies and procedures.

Antibody	Manufacturer company	Catalogue number	Protein molecular weight (kDa)	Gel %	Protein loaded (µg)	Blotting transfer time (min)	Blocking reagent	Primary antibody concentration	Secondary antibody concentration
Ser^{40} Nrf2	Abcam	ab76026	100	10	15	90	BSA 4%	1:5000	1:5000
Total Nrf2	Abcam	ab62352	100	12.5-15	12.5	90	BSA 4%	1:1500	1:5000
Thr^{287} CaMKII	Cell Signaling	12716	51	10	10	90	BSA 4%	1:2000	1:5000
Thr^{180}/Tyr^{182}-p38 MAPK	Cell Signaling	9211	43	7.5-10	10	90	Blotto 5%	1:3000	1:20,000
Ser^{9} GSK3β	Cell Signaling	5558	46	12.5-15	12.5	90	BSA 4%	1:1000	1:10,000
Catalase	Cell Signaling	14097	60	7.5-10	10	90	Blotto 5%	1:2000	1:5000
Keap1	Proteintech	10503-2-AP	70	10	7.5	90	BSA 4%	1:3000	1:10,000
GSR	Proteintech	18257-1-AP	52	10	8.5	90	Blotto 5%	1:2000	1:10,000
